# A dendritic nano-sized hexanuclear ruthenium(II) complex as a one- and two-photon luminescent tracking non-viral gene vector

**DOI:** 10.1038/srep10707

**Published:** 2015-07-17

**Authors:** Kangqiang Qiu, Bole Yu, Huaiyi Huang, Pingyu Zhang, Juanjuan Huang, Shanshan Zou, Yu Chen, Liangnian Ji, Hui Chao

**Affiliations:** 1 MOE Key Laboratory of Bioinorganic and Synthetic Chemistry, School of Chemistry and Chemical Engineering, Sun Yat-Sen University, Guangzhou 510275, P. R. China

## Abstract

Fluorescent tracking gene delivery could provide us with a better understanding of the critical steps in the transfection process. However, for *in vivo* tracking applications, a small diameter (<10 nm) is one of the rigorous requirements for tracking vectors. Herein, we have demonstrated a new paradigm for two-photon tracking gene delivery based on a dendritic nano-sized hexanuclear ruthenium(II) polypyridyl complex. Because this metallodendrimer has a multivalent periphery, the complex, which is 6.1 nm, showed high stability and excellent dispersibility and could stepwise condense DNA *in vitro*. With the outstanding photochemical properties of Ru(II) polypyridyl, this complex could track gene delivery *in vivo* using one- and two-photon imaging.

Gene therapy is a promising technique for treating human disease by the transfer of genetic material into specific cells within the patient^1^,^2^,^3^. Compared with viral vectors *in vivo*, nonviral gene delivery systems have gained immense importance in terms of safety^4^,^5^,^6^,^7^,^8^. As nonviral gene vectors, nanomaterials have been widely used to deliver genetic material *in vivo*^9^,^10^,^11^,^12^,^13^, and some of them can function as fluorescent tracking vectors^14^,^15^,^16^,^17^,^18^,^19^,^20^, which could provide us with a better understanding of the critical steps in the transfection process^21^. The tracking nanomaterials are usually labelled with organic dyes^14^,^15^, quantum dots^16^,^17^,^18^, carbon dots^19^ or metal complexes^20^. To apply these materials for tracking *in vivo* specifically, the small diameter (<10 nm) is one of the rigorous requirements for nanomaterials^22^,^23^,^24^,^25^. However, synthesizing small tracking nanomaterials generally requires multiple complicated reactions. Additionally, quantum dot-based nanomaterials contain toxic heavy metals, while small organic dyes suffer from small Stokes shifts and rapid photobleaching^26^,^27^,^28^. Even worse, the introduction of fluorescent materials may alter the delivery mechanisms and increase potential side-effects^29^.

Very recently, several efforts have been devoted to the development of multinuclear complexes that induce DNA condensation due to their greater variety of charge states and can be used as non-viral gene vectors^21^,^30^,^31^,^32^,^33^,^34^. As multinuclear complexes with metal fragments located within the dendrimers or at the periphery, metallodendrimers have applications in sensing, catalysis, biomedicine, and materials science^35^,^36^,^37^,^38^,^39^,^40^,^41^ based on specific properties, including highly controlled nanosize, tunable solubility and multivalent periphery^42^. However, using a Ru(II) polypyridyl metallodendrimer-based vector to track intracellular gene delivery has been rarely explored. Moreover, Ru(II) polypyridyl complexes have outstanding photochemical properties, such as high photostability, large Stokes shifts, great one- and two-photon luminescence properties and large two-photon absorption cross sections, which have made these complexes promising luminescent tools for two-photon tracking studies^43^,^44^,^45^,^46^. Therefore, we attempted to employ a dendritic luminophore with proton buffering capacity polybenzimidazole ligands^30^,^31^ as a tracking non-viral gene vector. This complex would allow us to observe gene condensing *in vitro* and track gene delivering *in vivo*.

Herein, a dendritic hexanuclear Ru(II) polypyridyl complex based on polybenzimidazole ligands, **Ru**_**6**_**L** (Fig. 1), was synthesized and developed as a one- and two-photon luminescent tracking non-viral gene vector. The photophysical properties and the size of **Ru**_**6**_**L** were characterized. After studying the interactions of **Ru**_**6**_**L** with DNA and the DNA condensation mechanism *in vitro*, the cellular uptake of both the complex and the **Ru**_**6**_**L**-DNA particles was investigated. Then, the complex was used as a one- and two-photon luminescent tracking non-viral gene vector. Finally, the transfection efficiency and cytotoxicity of the DNA condensates were evaluated by luciferase assays and MTT assays.

## Results and Discussion

**Figure 1:**
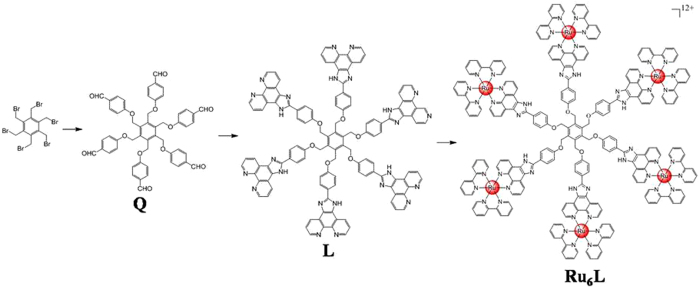


The synthesis of the ligand and complex was outlined in Fig. 1. The bridging ligand 1,2,3,4,5,6-hexakis{4-((1,10-phenanthroline-[5,6-d]imidazol-2-yl)-phenoxy)methyl}benzene (**L**) was obtained at a 58% yield through condensation of 1,10-phenanthroline-5,6-dione with 1,2,3,4,5,6-hexakis[(4-formylphenoxy)methyl]benzene in refluxing glacial acetic acid. The ^1^H NMR spectrum of ligand **L** was not obtained due to its poor solubility in common NMR solvents. Complex {[Ru(bpy)_2_]_6_L}(ClO_4_)_12_ (**Ru**_**6**_**L**) was prepared by refluxing Ru(bpy)_2_Cl_2_.2H_2_O and **L** in ethylene glycol and isolated as a ClO_4_^–^ salt, with a 62% yield. This complex was characterized by ^1^H NMR spectroscopy, IR spectroscopy, ESI-MS and elemental analysis (Figure S1-S3).

**Figure 2:**
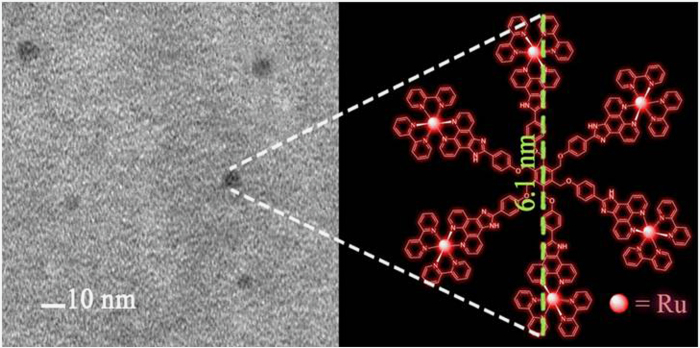


The photophysical data of **Ru**_**6**_**L** are summarized in Table S1. The absorption band at approximately 400–550 nm was assigned to metal-ligand charge transfer (MLCT) absorption (Figure S4). The maximum emission wavelength (λ_em_) was 612 nm, with a lifetime of 110 ns. The emission quantum yield was 0.027, and the largest two-photon absorption (TPA) cross-section was 175 GM at 830 nm with reference to rhodamine B (Figure S5). The complex was a perfectly monodisperse nanoparticle with 12 positive charges derived from Ru^2+^ ions (Figure S6). The diameter of **Ru**_**6**_**L** was determined to be 6.1 nm by TEM (Fig. 2), which was similar to the hydrodynamic diameter 6.7 ± 0.3 nm determined by DLS (Figure S6).

**Figure 3:**
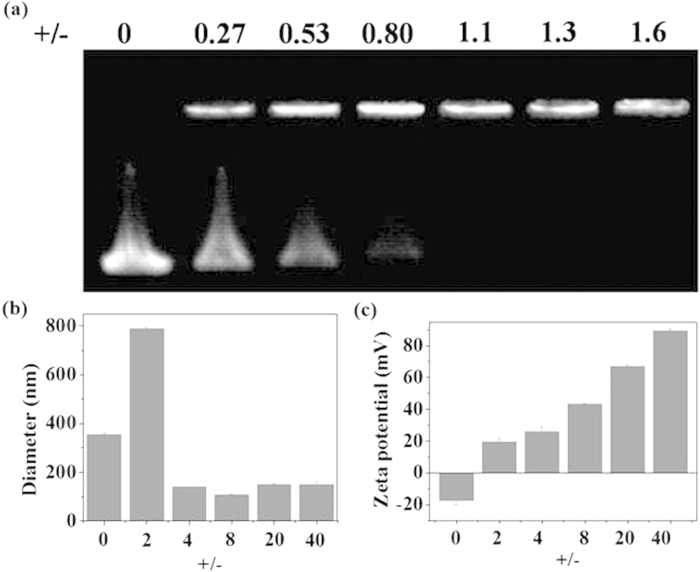
(**a**) Agarose gel electrophoresis of pBR 322 DNA (45 μM) after incubation with Ru6L at various +/− ratios in aqueous solution; (**b**) Hydrodynamic diameter and (**c**) zeta potential of the pBR 322 DNA (1.5 μM) incubation with Ru6L at various +/− ratios in an aqueous solution,as determined by DLS.

We investigated the complex’s ability to condense DNA. The first study examined the migration of **Ru**_**6**_**L**-pBR 322 DNA by gel retardation assays with a load of 45 μM (bp) DNA in each well. As shown in Fig. 3, with increasing the concentrations of **Ru**_**6**_**L** (0-6 μM), pBR 322 DNA gradually diminished and was retained in the well at a +/− ratio (total positive charge/total negative charge) of 1.1. The primary DNA condensation-driving forces were considered electrostatic interactions by the multivalent periphery of **Ru**_**6**_**L**. The DNA binding ability was determined by a DNA titration approach with CT-DNA (calf thymus DNA). The binding constant was calculated to be 1.75 × 10^5^ M^−1^ (Figure S7).

**Figure 4:**
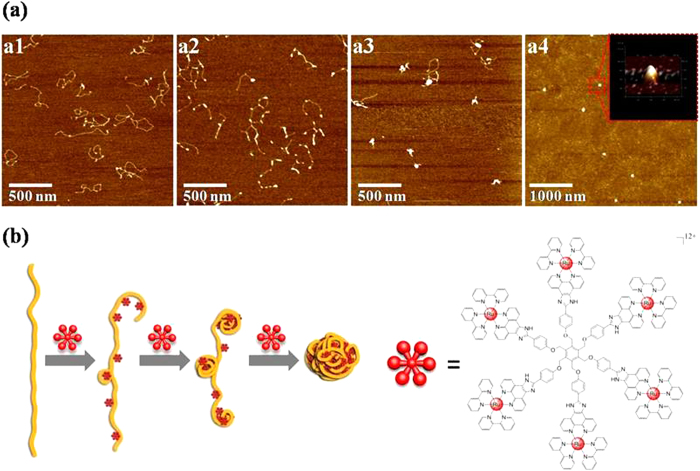
(**a**) AFM images of the condensation process, starting with2 kb DNA (1.5 μM), induced by incubation with Ru6L at +/− ratios of 1 (a1), 2 (a2), 4 (a3), and 20 (a4), respectively. (**b**) Schematic representation of the stepwise DNA compaction by **Ru**_**6**_**L**.

The average hydrodynamic diameter of **Ru**_**6**_**L**-pBR 322 DNA particles at various +/− ratios in aqueous solution is shown in Fig. 3. The particle diameter was stable at approximately 140 nm when the +/− ratio was 4 or higher, while the zeta potential increased as the +/− ratio increased. To visually investigate the condensation process, we performed AFM of the **Ru**_**6**_**L**-2kb DNA particles at various +/− ratios (Fig. 4). When the +/− ratio was 1, local DNA bending, long-range cross-links and micro-loops were observed. Upon increasing the +/− ratio to 2, small particles were observed at the middle or the tail of the DNA duplex. As the +/− ratio increased, highly condensed DNA molecules were observed, and they condensed into compact hemispherical structures at a +/− ratio of 20. **Ru**_**6**_**L**-pBR 322 DNA particles at a +/− ratio of 20 were also observed as well-distributed DNA particles with a diameter from 50 to 90 nm (Figure S8).

In addition, we performed a DNA nuclease-catalyzed biodegradation assay (Figure S9a), a photocleavage assay (Figure S9b) and a continuous irradiation assay (Figure S10) with **Ru**_**6**_**L**. The results showed that **Ru**_**6**_**L** displayed no photocleavage or photoreactivity toward DNA; in contrast, it protected DNA against nuclease-catalyzed biodegradation.

Before performing cellular uptake studies of **Ru**_**6**_**L**-DNA particles, we investigated the mechanism of cellular uptake of **Ru**_**6**_**L** using confocal microscopy (Figure S11). Generally speaking, the cellular uptake of small molecules can occur through energy-independent (facilitated diffusion, passive diffusion) and energy-dependent (endocytosis, active transport) pathways. To determine whether **Ru**_**6**_**L** entered the cell via an energy-independent or energy-dependent transport pathway, HeLa cells were either incubated with **Ru**_**6**_**L** at 4 °C or pretreated with the metabolic inhibitors 2-deoxy-D-glucose and oligomycin. Figure S11b and S11c show that cellular luminescence was significantly suppressed in cases when the cells were incubated with the complex at 4 °C or pretreated with the metabolic inhibitors, indicating that **Ru**_**6**_**L** uptake followed an energy-dependent pathway. Endocytosis is well known as the most common energy-dependent pathway by which eukaryotic cells uptake extracellular materials. Endocytosis is also affected by temperature or adenosine triphosphate (ATP). We used the endocytic inhibitors chloroquine and NH_4_Cl to examine the role of this pathway in **Ru**_**6**_**L** uptake. As observed from the relative intensities and location of **Ru**_**6**_**L** in the cells after treatment with these inhibitors (Figure S11d and S11e), they had no effect on the cellular uptake of **Ru**_**6**_**L**, which indicates that **Ru**_**6**_**L** is not taken up by living cells *via* an endocytosis pathway. This finding is not surprising because cellular uptake mechanisms are generally complex and diverse.

**Figure 5:**
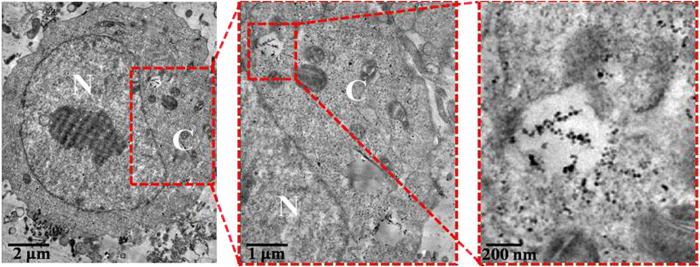
Cellular uptake and intracellular localization of Ru6L-pEGFP DNA particles at the +/− ratio of 20 monitored, as by TEM. The DNA concentration was 1.5 μM, N: nucleus, C: cytoplasm.

Flow cytometry was used to quantify the intracellular uptake of the DNA particles at the +/− ratio of 20 (Figure S12). The result indicated that most of cells took up the DNA particles after a 4 h incubation in serum-free DMEM. We examined the localization of DNA particles in HeLa cells by TEM. TEM micrographs confirmed the uptake of the particles by endocytosis and showed that the DNA particles made HeLa cell form an endosome after they entered the HeLa cells (Fig. 5). Particles were also found in the cytoplasm, meaning that the particles successfully escaped from endosomes, which was important for transfection. The particle size was approximately 50 nm, as measured by AFM. The mechanism of cellular uptake of the DNA particles was also studied (Figure S13). The cellular luminescence of Figure S13b which incubated with the particles at 4 °C was significantly suppressed, it means the particles entered the cell *via* an energy-independent transport pathway. After treatment with the metabolic inhibitors (Figure S13c) and the endocytic inhibitors (Figure S13d and S13e), no effect was found in the cells which treated with the metabolic inhibitors, but the cellular luminescence which treated with the endocytic inhibitors was suppressed, the results indicated that **Ru**_**6**_**L**-pEGFP DNA particles were taken up by living cells *via* an endocytosis pathway.

**Figure 6:**
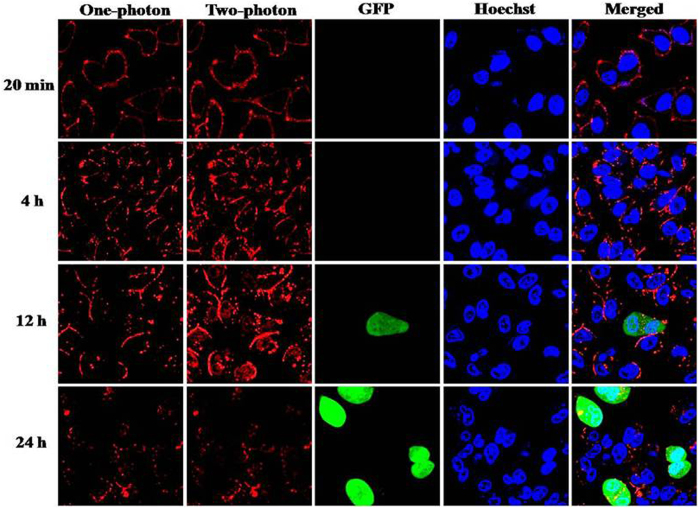
Time-dependent confocal microscopy images of entry and transportation of **Ru6L**-pEGFP DNA particles at the +/− ratio of 20 in HeLa cells. The DNA concentration was 1.5 μM. The red luminescence is **Ru**_**6**_**L**, the blue florescence is Hoechst 33258 and the green florescence is GFP.

To investigate the intracellular behaviors of **Ru**_**6**_**L**-pEGFP DNA particles, one- and two-photon fluorescence microscopy was used to monitor the time-dependent transport and transfection of pEGFP DNA plasmids that were condensed by **Ru**_**6**_**L** (Fig. 6). We stained the nuclei of HeLa cells with Hoechst 33258; then, the DNA particles were added to the cells. In 20 min, the **Ru**_**6**_**L**-DNA particles attached to the cell membrane quickly due to their high positive zeta potential. After 4 h, particles were found in the cytoplasm. Then, the culture medium was replaced with fresh DMEM containing 10% fetal bovine serum (FBS). After another 12 h, EGFP expression was detected, and higher EGFP expression was found after 24 h. Similar behaviors were observed under one-photon and two-photon excitation. In addition, due to the two-photon absorption, the background signal was strongly suppressed, and a higher resolution image was obtained using two-photon confocal laser scanning microscopy.

The most commonly used method for intracellular plasmid trafficking is the fluorescent labeling of non-viral vectors with organic dyes^14^,^15^. It should be pointed out that to track the dynamic changes during a specific period of time, the dye must possess improved photostability and must be photostable under continual irradiation with light from fluorescent microscopes. However, most organic dyes have notable shortcomings, including poor water solubility, high toxicity to living cells and poor photostability. Organic dyes may also cause extensive cellular damage and unwanted background signal due the ultraviolet (UV) radiation required for their excitation and small Stokes shifts^26^,^27^. The short excitation wavelengths (<650 nm) also inhibit the use of these materials in thick tissues or live animals due to the resultant short penetration depth^47^. In addition, the introduction of dyes may alter the delivery mechanisms and have increased side-effects^29^. The use of **Ru**_**6**_**L** with intrinsic two-photon luminescence as DNA carriers is an attractive solution to these problems because this complex exhibits near infrared (NIR) excitation wavelengths^48^.

**Figure 7:**
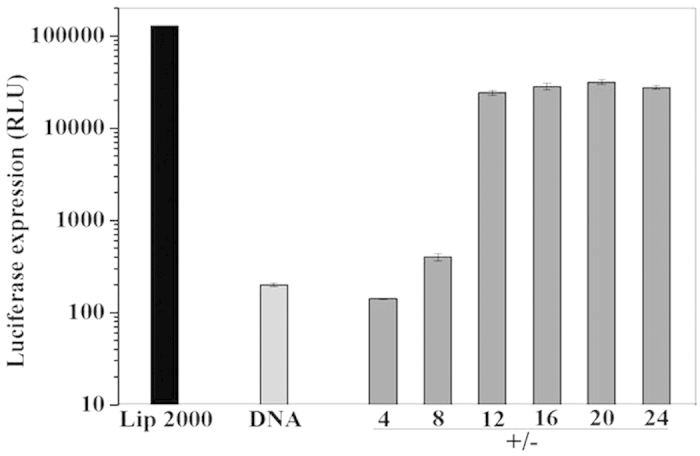
Transfection efficiencies of Ru6L-pGL3 DNA particles in HeLa cells, as determined by luciferase assays; the DNA concentration was 1.5 μM. As a control, DNAand lipofectamine 2000 were also investigated.

We determined the relative transfection efficiency of this nonviral system by luciferase assays, and plasmid pGL3 was used a control vector (Fig. 7). As a control, applying DNA only resulted in a low level of luciferase expression. The luciferase expression was increased when **Ru**_**6**_**L** was used. The luciferase expression was stable when the +/− ratios of the particles ranged from 12 to 24, and the highest luciferase expression was in response to the +/− ratio of 20.

**Figure 8:**
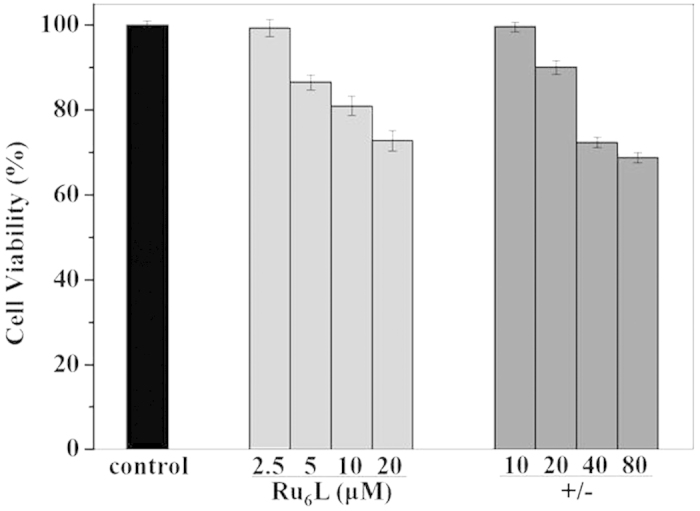
The cell viability of Ru6L and Ru6L-pEGFP DNA particles in HeLa cells, as determined by MTT assays; the DNA concentration was 1.5 μM.

Cytotoxicity of non-viral gene vectors is one of the major concerns of gene delivery. Therefore, we examined the viability of HeLa cells treated with the complex and **Ru**_**6**_**L**-pEGFP DNA particles (Fig. 8). With increasing concentrations, the viability of the HeLa cells decreased slowly for both the complex and the particles. Although **Ru**_**6**_**L** exhibited the related cytotoxicity as the concentration increased, the viability of HeLa cells was more than 80% at a concentration of 5 μM. The results indicated that this transfection system had relatively low cytotoxicity.

## Conclusion

In the present study, we have designed and synthesized a dendritic nano-sized hexanuclear Ru(II) polypyridyl complex (**Ru**_**6**_**L**) based on polybenzimidazole ligands. This complex has a multivalent periphery and a nanosize of 6.1 nm. Gel retardation assay, TEM, AFM and dynamic light scattering studies show that **Ru**_**6**_**L** exhibits high stability and excellent dispersibility and could stepwise condense DNA *in vitro*. More interestingly, **Ru**_**6**_**L** was applied as a non-viral gene vector for tracking DNA delivery in live cells using one- and two-photon fluorescence microscopes. With two-photon microscopy, a high signal-to-noise contrast was achieved by irradiation with an 830 nm laser. Our work provides new insights into improving real-time tracking during gene delivery and transfection as well as important information for the design of multifunctional non-viral vectors.

## Methods

All reagents were purchased from commercial sources and used without further purification unless otherwise specified. The plasmid pBR 322 DNA was obtained from MBI Fermentas, the plasmid pEGFP DNA was purchased from Clontech, and the plasmid pGL3 control vector and luciferase kid were obtained from Promega. Unless otherwise stated, the DNA concentrations are expressed in base pairs. All samples were prepared using distilled water that had been passed through a Millipore-Q ultra-purification system. The compounds 1,10-phenanthroline-5,6-dione^49^ and [Ru(bpy)_2_]Cl_2_.2H_2_O^50^ were prepared according to literature methods. The complex was dissolved in DMSO prior to the experiments. Then, the calculated quantities of the complex solutions were added to the appropriate medium to yield a final DMSO concentration of less than 1% (v/v).

Microanalyses (C, H and N) were performed with a vario EL cube elemental analyzer. Infrared spectra were obtained with a Nicolet 170SX-FTIR spectrophotometer and KBr discs. Electrospray ionization mass spectra (ESI-MS) were recorded on an LCQ system (Finnigan MAT, USA).Fast atom bombardment mass spectrometry (FAB-MS) was recorded using a VG ZAB-HS. ^1^H NMR spectra were recorded on a Varian-500 spectrometer at 25 °C. All chemical shifts are given relative to tetramethylsilane (TMS). The UV-Vis spectra were recorded on a Perkin-Elmer Lambda 850 spectrophotometer. Emission spectra were recorded on a Perkin-Elmer LS 55 spectrophotometer at room temperature (25 °C). Time-resolved emission measurements were conducted on an FLS 920 combined fluorescence-lifetime and steady-state spectrometer. Quantum yields of luminescence at room temperature (25 °C) were calculated according to literature procedures using [Ru(bpy)_3_]^2+^ (*φ* = 0.028 in aerated aqueous solution) as the reference emitter^51^. All date were processed using the Origin 8 software package.

Atomic force microscopy (AFM) images were obtained in air at room temperature with an SPA400 atomic force microscope unit and an SPI3800N control station (Seiko Instruments) operated in the tapping mode. Probes were made of a single silicon crystal with a cantilever length of 129 mm and a spring constant of 33–62N/m (OMCLAC160TS-W2, Olympus). Dynamic light scattering and zeta potential experiments were performed using dynamic laser light scattering equipment (DLS, Brooken Haven BI-200SM). Transmission electron micrographs were obtained with a JEM100CX electron microscope.

### Synthesis of 1,2,3,4,5,6-hexakis[(4-formylphenoxy)methyl]benzene (Q)

A mixture of 4-hydroxybenzaldehyde (0.50 g, 4.09 mmol), 1,2,3,4,5,6-hexakis(bromomethyl)benzene (0.30 g, 0.47 mmol), and K_2_CO_3_ (0.60 g, 4.35 mmol) in DMF (30 mL) was heated to 80 °C for 24 h under nitrogen. After cooling to room temperature, the solution was diluted with 300 mL of water. The mixture was filtered, and the solid was washed successively with boiling MeOH, CHCl_3_ and Et_2_O, affording the desired product as a white solid. Yield: 0.21 g (50%). Anal. Calcd. for C_54_H_42_O_12_: C, 73.46; H, 4.79. Found: C, 73.24; H, 4.80. IR *ν*_max_ (KBr, cm^−1^): 1689 (CHO).^1^H NMR (500 MHz, d_6_-DMSO) δ 9.76 (s, 6H), 7.70 (d, *J* = 15 Hz, 12H), 7.04 (d, *J* = 15 Hz, 12H), 5.42 (s, 12H). ESI-MS: m/z = 905.4 [M+Na]^+^.

### Synthesis of 1,2,3,4,5,6-hexakis{4-((1,10-phenanthroline-[5,6-d]imidazol-2-yl)- phenoxy)methyl}benzene (L)

A mixture of **Q**(0.15 g, 0.17 mmol), 1,10-phenanthroline-5,6-dione (0.30 g, 1.42 mmol) and NH_4_OAc (2.00 g, 25.64 mmol) in glacial acetic acid (20 mL) was heated to 130 °C for 8 h, yielding a suspension. The reaction mixture was filtered while hot, and the solid was washed successively with EtOH, hot DMF and Et_2_O, affording the desired product as a yellow solid. Yield: 0.20 g (58%). Anal. Calcd. for C_126_H_78_N_24_O_6_: C, 74.77; H, 3.88; N, 16.61. Found: C, 74.55; H, 4.16; N, 16.43. IR *ν*_max_ (KBr, cm^−1^): 3392 (N-H). FAB-MS: m/z = 2025 [M+1]. The ^1^H NMR spectrum was not obtained due to its poor solubility in common NMR solvents.

### Synthesis of complex {[Ru(bpy)_2_]_6_L}(ClO_4_)_12_ (Ru_6_L)

A mixture of **L** (0.075 g, 0.037 mmol) and Ru(bpy)_2_Cl_2_.2H_2_O (0.15 g, 0.29 mmol) in ethylene glycol (10 mL) was heated to 180 °C for 14 h under nitrogen to give a clear deep-red solution. The cooled reaction mixture was diluted with water (15 mL). Saturated aqueous sodium perchlorate solution was added under vigorous stirring, and the solution filtered. The dark red solid was collected, washed with small amounts of water and diethyl ether, dried under vacuum, and purified by column chromatography on alumina using acetonitrile-ethanol as the elutant. The solvent was removed under reduced pressure, and red microcrystals were obtained. Yield: 0.13 g (62%). Anal. Calcd. for C_246_H_174_Cl_12_N_48_O_54_Ru_6_: C, 51.85; H, 3.08; N, 11.80. Found: C, 51.64; H, 3.37; N, 11.57.IR *ν*_max_ (KBr, cm^−1^): 3360 (N-H) and 1087 (ClO_4_^−^). ESI-MS: m/z = 423.6 [M-6ClO_4_-6H]^12+^, 438.8 [M-9ClO_4_-6H]^11+^, 453.8 [M-7ClO_4_-6H]^11+^, 481.9 [M-9ClO_4_-6H]^10+^, 500.8 [M-12ClO_4_+4H]^9+^, 512.0 [M-11ClO_4_-5H]^9+^, 554.5 [M-7ClO_4_-6H]^9+^, 574.9 [M-11ClO_4_-4H]^8+^, 600.5 [M-9ClO_4_+2H]^8+^, 642.7 [M-12ClO_4_-5H]^7+^, 657.6 [M-11ClO_4_]^7+^, 672.4 [M-10ClO_4_-3H]^7+^, 750.5 [M-12ClO_4_-H]^6+^, 766.5 [M-11ClO_4_-4H]^6+^, 782.8 [M-10ClO_4_-6H]^6+^, 799.4 [M-9ClO_4_-6H]^6+^, 815.5 [M-8ClO_4_-6H]^6+^, 832.8 [M-7ClO_4_-6H]^6+^, 901.3 [M-12ClO_4_+2H]^5+^, 921.0 [M-11ClO_4_-H]^5+^, 940.5 [M-10ClO_4_]^5+^, 959.0 [M-9ClO_4_-6H]^5+^, 979.7 [M-8ClO_4_-3H]^5+^. ^1^H NMR (500 MHz, d_6_-DMSO) δ 9.10 (bs, 12H), 8.81 (q, *J* = 5 Hz, 24H), 8.21 (d, *J* = 10 Hz, 12H), 8.20 (t, *J* = 7.5 Hz, 12H), 8.04 (t, *J* = 7.5 Hz, 12H), 7.95 (s, 12H), 7.82 (d, *J* = 5 Hz, 24H), 7.54–7.59 (m, 24H), 7.28 (q, *J* = 5 Hz, 24H), 5.56 (bs, 12H).

### Determining the size of the complex

The complex **Ru**_**6**_**L** was dissolved in CH_3_CN at a concentration of 5 μM. The solution was drop cast onto a carbon-coated Cu grid, and the excess solution was absorbed by filter paper to avoid aggregation. The TEM image of the drop cast sample was taken with a JEM100CX electron microscope.

Dynamic laser light scattering equipment was used to determine the average hydrodynamic diameter and the zeta potential of **Ru**_**6**_**L** at a concentration of 5 μM in distilled water that had been passed through a Millipore-Q ultra-purification system. Typically, 5 runs were measured for the solution, and the average of all runs is reported.

### Determination of two-photon absorption cross-sections

The two-photon absorption (TPA) spectra of complex were determined over a broad spectral region by a two-photon induced luminescence (TPL) method relative to Rhodamine B in methanol as the standard. The two-photon luminescence data were acquired using an Opolette^TM^ 355II (pulse width ≤ 100 fs, 80 MHz repetition rate, tuning range 730–890 nm, Spectra Physics, Inc., USA). Two-photon luminescence measurements were performed in fluorometric quartz cuvettes. The experimental luminescence excitation and detection conditions were conducted with negligible reabsorption processes, which can affect TPA measurements. The quadratic dependence of the two-photon induced luminescence intensity on the excitation power was verified at an excitation wavelength of 830 nm. The two-photon absorption cross-section of the complex was calculated at each wavelength according to Equation (1)^52^:


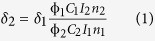


where *I* is the integrated luminescence intensity, *C* is the concentration, *n* is the refractive index, and *φ* is the quantum yield. The subscript ‘1’ refers to the reference samples, and ‘2’ for the experimental samples.

### Preparation of DNA particles

The DNA particles were prepared by incubating the mixtures containing DNA and **Ru**_**6**_**L** at the given +/− ratios in 50 mM Tris-HCl (Tris = Tris(hydroxymethyl) aminomethane) solution (pH = 7.4) or in cell culture, followed by vortexing for 30 min to allow equilibration at room temperature.

### Gel retardation assay

Negative supercoiled pBR 322 DNA was treated with **Ru**_**6**_**L** in 50 mM Tris-HCl solution (pH = 7.4), and the solutions were analyzed by electrophoresis for 1.5 h at 75 V on a 1% agarose gel in TBE buffer (89 mM Tris-borate acid, 2 mM EDTA, pH = 8.3). The gel was stained with 1 μg/mL ethidium bromide (EB) and photographed on an Alpha Innotech IS-5500 fluorescence chemiluminescence and visible imaging system.

### Dynamic light scattering and zeta potential assay

Dynamic laser light scattering equipment was used to determine the average hydrodynamic diameter and the zeta potential of **Ru**_**6**_**L**-pBR 322 DNA particles at various +/− ratios in 50 mM Tris-HCl solution (pH = 7.4). Typically, 5 runs were measured for each solution, with the average of all the runs reported.

### AFM imaging

The 2kb DNA fragment was generated by PCR using pBR 322 plasmid DNA as a template^53^. The condensation process of **Ru**_**6**_**L**-2kb DNA and the morphologies **Ru**_**6**_**L**-pBR 322 DNA were examined with AFM. Samples were dropwise-added (10 μL) onto a mica substrate, which was freshly cleaved by pulling off the top sheets with tape. One min later, the substrate was spin coated (1400 rpm, 30 s) and rinsed with 20 μL of distilled water. AFM images were obtained in air at room temperature. The images were captured in a 256 × 256 pixels format and analyzed with the software accompanying the imaging module.

### Cell culture and cell viability assay

HeLa cells were maintained as monolayer cultures in DMEM supplemented with 10% fetal bovine serum (FBS) at 37 °C under 5% CO_2_. Exponentially grown HeLa cells were seeded in triplicate onto 96-well plates at 1 × 10^4^ cells/well. After incubation for 24 h, the cells were treated with increasing concentrations of the tested complex and **Ru**_**6**_**L**-pEGFP DNA particles for 24 h. To stain the viable cells, 20 μL of MTT (5 mg/mL) was added to each well. The cells were then incubated for 4 h at 37 °C. After the medium was carefully aspirated without disturbing the formed crystals, the dye was dissolved in 200 μL of DMSO. A Tecan Infinite M200 monochromator-based multifunction microplate reader was used to measure the optical density of each well, with background subtraction at 590 nm. The cell survival rate in the control wells without **Ru**_**6**_**L** solutions were considered as 100% cell survival.

### The mechanism of cellular uptake of Ru6L

The cells were trypsinized, counted, and adjusted to 1 × 10^5^ cells mL^−1^ and 1 mL was added to five 35-mm^2^ Petri dishes (MatTek, USA) for laser confocal microscopy. After 24 h, the first dish was incubated with 5 μM **Ru**_**6**_**L** at 37 °C for 2 h. The second dish was incubated with 5 μM **Ru**_**6**_**L** at 4 °C for 2 h. The third dish was pretreated with 50 mM 2-deoxy-D-glucose and 5 μM oligomycin in PBS for 1 h at 37 °C and then incubated with 5 μM **Ru**_**6**_**L** at 37 °C for 2 h. The fourth and the fifth dishes were pretreated with endocytic inhibitors NH_4_Cl (50 mM), and chloroquine (50 μM) for 30 min respectively, and then incubated with 5 μM **Ru**_**6**_**L** at 37 °C for 2 h.

After being washed with fresh PBS (pH = 7.0) three times, the cells were imaged on a Zeiss LSM 710 NLO confocal microscope (63 × /NA 1.4 oil immersion objective). For two-photon images, the excitation wavelength of the laser was 830 nm for **Ru**_**6**_**L** and the emission spectra were integrated over 580-630 nm (single channel).

### Cellular uptake of Ru6L-DNA particles

The cells were trypsinized, counted, and adjusted to 1 × 10^4^ cells mL^−1^, and 1 mL of the cell solution was added to each plate. After 24 h, the cell culture medium was replaced with 800 μL serum-free DMEM. **Ru**_**6**_**L**-pEGFP DNA particles at the +/− ratio of 20, containing 1 μg pEGFP DNA in 200 μL serum-free DMEM, was added to the cells, and the cells were incubated at 37 °C for 4 h.

For flow cytometry, the cells were washed with PBS three times, trypsinized and centrifuged in PBS. Cells were harvested, and single cell suspensions in 0.5 mL PBS were prepared and subjected to flow cytometric analysis. A flow cytometer (Coulter Co. USA) was used to measure the fluorescent intensity, with excitation at 488 nm.

For TEM imaging analysis, cell processing was performed *in situ*, without displacement from the culture dish. Cells were fixed in 0.1 M PBS containing 2.5% glutaraldehyde and 4% paraformaldehyde for 1 h, rinsed with distilled water, stained with 0.5% uranyl acetate for 1 h, dehydrated in a graded series of ethanol (30, 60, 70, 90 and 100%), and embedded in epoxy resin. The resin was polymerized at 60 °C for 48 h. Ultrathin sections (50–75 nm) obtained with an LKB ultramicrotome were stained with 2% aqueous uranyl acetate and 2% aqueous lead citrate.

### The mechanism of cellular uptake of Ru6L-DNA particles

The cells were trypsinized, counted, and adjusted to 1 × 10^5^ cells mL^−1^ and 1 mL was added to five 35-mm^2^ Petri dishes (MatTek, USA) for laser confocal microscopy. After 24 h, the first dish was incubated with **Ru**_**6**_**L**-pEGFP DNA particles at the +/− ratio of 20 at 37 °C for 4 h. The second dish was incubated with **Ru**_**6**_**L**-pEGFP DNA particles at the +/− ratio of 20 at 4 °C for 4 h. The third dish was pretreated with 50 mM 2-deoxy-D-glucose and 5 μM oligomycin in PBS for 1 h at 37 °C and then incubated with **Ru**_**6**_**L**-pEGFP DNA particles at the +/− ratio of 20 at 37 °C for 4 h. The fourth and the fifth dishes were pretreated with endocytic inhibitors NH_4_Cl (50 mM), and chloroquine (50 μM) for 30 min respectively, and then incubated with **Ru**_**6**_**L**-pEGFP DNA particles at the +/− ratio of 20 at 37 °C for 4 h.

After being washed with fresh PBS (pH = 7.0) three times, the cells were imaged on a Zeiss LSM 710 NLO confocal microscope (63 × /NA 1.4 oil immersion objective). For two-photon images, the excitation wavelength of the laser was 830 nm for **Ru**_**6**_**L** and the emission spectra were integrated over 580-630 nm (single channel).

### One- and two-photon luminescent imaging

The cells were trypsinized, counted, and adjusted to 1 × 10^5^ cells mL^−1^, and 1 mL of the cell suspension was added to a 35-mm^2^ Petri dish (MatTek, USA) for laser confocal microscopy. After 24 h, the cell culture medium was replaced with 800 μL serum-free DMEM. **Ru**_**6**_**L**-pEGFP DNA particles at the +/− ratio of 20, containing 1 μg pEGFP DNA in 200 μL serum-fee DMEM, was added to the cells, and the cells were incubated at 37 °C for 4 h. Then, the medium was replaced with fresh DMEM containing 10% FBS, and the cells were incubated for various timed (20 min and 4, 12, and 24 h).

After being washed with fresh PBS (pH = 7.0) three times, the cells were imaged on a Zeiss LSM 710 NLO confocal microscope (63 × /NA 1.4 oil immersion objective). The excitation wavelength of the laser was 488 nm, and the emission spectra were integrated over 580-630 nm (single channel). For two-photon images, the excitation wavelength of the laser was 830 nm for **Ru**_**6**_**L**.

### Luciferase assay

HeLa cells were seeded onto a 96-well cell-culture plate at a cell density of 1 × 10^4^ cells per well and then incubated for 24 h. Cells were washed with PBS three times and then cultured with serum-free DMEM. **Ru**_**6**_**L**-pGL3 DNA control vector particles with increasing concentrations of the tested complex containing 0.2 μg plasmid were added to the cells, and the cells were incubated at 37 °C for 4 h. The medium was then replaced with fresh DMEM with 10% FBS, and the cells were incubated for an additional 24 h. Cells were washed with PBS, harvested and treated for 30 min at 4 °C with end-over-end rotation in lysis buffer (50 mM Tris-HCl, pH = 7.5, 150 mM NaCl, 2% Triton X-100, 2% NP40). The luciferase assay was performed according to the manufacturer’s protocol (Promega). Relative light units (RLUs) were measured with Varioskan Flash (Thermo Scientific, USA) and a GloMax^TM^ 96 microplate luminometer (Promega, USA).

## Additional Information

**How to cite this article**: Qiu, K. *et al.* A dendritic nano-sized hexanuclear ruthenium(II) complex as a one- and two-photon luminescent tracking non-viral gene vector. *Sci. Rep.* **5**, 10707; doi: 10.1038/srep10707 (2015).

## Supplementary Material

Supplementary Information
