# Supplementary material for: A dendritic nano-sized hexanuclear ruthenium(II) complex as a one- and two-photon luminescent tracking non-viral gene vector
Source: Sci Rep. 2015 Jul 17;5:10707. doi: 10.1038/srep10707 (PMC4505312; doi:10.1038/srep10707)
Supplement: Supplementary Information [file srep10707-s1.doc]

Supporting Information

**A dendritic nano-sized hexanuclear ruthenium(II) complex as a one- and two-photon luminescent tracking non-viral gene vector**

**Kangqiang Qiu, Bole Yu, Huaiyi Huang, Pingyu Zhang, Juanjuan Huang,**

**Shanshan Zou, Yu Chen, Liangnian Ji & Hui Chao***

*MOE Key Laboratory of Bioinorganic and Synthetic Chemistry, School of Chemistry and Chemical Engineering, Sun Yat-Sen University, Guangzhou 510275, P. R. China*

Tel: +86 20 84110613; Fax: +86 20 84112245; Email: ceschh@mail.sysu.edu.cn

**Table of Contents**

DNA binding assay......................................................................................................S2

DNase-I protection assay............................................................................................S2

DNA photocleavage assay..........................................................................................S3

Continuous irradiation in presence of DNA.................................................................S3

**Table S1** Photophysical data of **Ru_6_L**.........................................................................S4

**Figure S1** ^1^H NMR spectrum of **Q**.............................................................................S5

**Figure S2** ESI-MS spectrum of **Ru_6_L**.........................................................................S6

**Figure S3** ^1^H NMR spectrum of **Ru_6_L**.......................................................................S7

**Figure S4** Absorption spectras and emission spectra of **Ru_6_L**....................................S8

**Figure S5** Two-photon absorption properties of **Ru_6_L**...............................................S9

**Figure S6** Hydrodynamic diameter and zeta potential of **Ru_6_L**................................S10

**Figure S7** The DNA binding ability of **Ru_6_L**..........................................................S11

**Figure S8** AFM image of **Ru_6_L**-pBR 322 DNA at the +/- ratio of 20......................S12

**Figure S9** DNase-I protection and DNA photocleavage of **Ru_6_L**-pBR 322 DNA..S13

**Figure S10** Continuous irradiation............................................................................S14

**Figure S11** The mechanism of cellular uptake of **Ru_6_L**………....………….….......S15

**Figure S12** Quantitative flow cytometry results………...………………….….......S16

**Figure S13** The mechanism of cellular uptake of **Ru_6_L**-DNA particles…………...S17

References……………………………………….…………….…………................S18

**DNA binding assay.** The DNA-binding experiment was performed at room temperature. UV-Vis spectra were recorded on a Perkin-Elmer Lambda 850 spectrophotometer and the spectroscopic titration was carried out in buffer A (5 mM Tris-HCl, 50 mM NaCl, pH = 7.2). The DNA concentration per nucleotide was determined by absorption spectroscopy using the molar absorption coefficient (6600 M^-1^cm^-1^) at 260 nm^1^. A solution of CT-DNA in the buffer gave a ratio of the UV absorbance at 260 and 280 nm of 1.8-1.9:1, indicating that the DNA was sufficiently free of protein^2^.

The absorption titration experiments were performed by maintaining a **Ru_6_L** concentration (5 μM) and by varying the nucleotide concentration (0-45 μM) in buffer. The mixed solutions were allowed to incubate for 5 min before the absorption spectra were recorded. The intrinsic binding constants *K_b_* to DNA were determined using Equation (2) ^3^:

[DNA]/(*ε*_a_ - *ε*_f_) = [DNA]/(*ε*_b_ - *ε*_f_) + 1/*K*_b_(*ε*_b_ - *ε*_f_) (2)

where [DNA] is the concentration of DNA in base pairs, the apparent absorption coefficients 𝜀*_a_*, 𝜀*_f_* and 𝜀*_b_* correspond to A_obsd_/[Ru], the extinction coefficient for the free ruthenium complex, and the extinction coefficient for the ruthenium complex in the fully bound form, respectively. A plot of [DNA]/[*ε*_a_-*ε*_f_] versus [DNA] gave a slope 1/[*ε*_a_-*ε*_f_] and Y intercept equal to 1/K_b_[*ε*_b_-*ε*_f_], respectively. The intrinsic binding constant K_b_ is given by the ratio of the slope to the intercept.

**DNase-I protection assay. Ru_6_L**-pBR 322 DNA particles at various +/- ratios, containing 1 μg pBR 322 DNA and 1 μg free pBR 322 DNA were incubated at 37 °C for 30 min in the presence of 1 unit of DNase-I in the digestion buffer consisting of 50 mM Tris-HCl (pH = 7.4), 2.5 mM MgCl_2_ and 0.5 mM CaCl_2_. After DNase-I digestion, the solution was treated with 5 μL of 250 mM EDTA (pH = 8.0) for 10 min to inactivate DNase-I and then mixed with sodium dodecyl sulfate (SDS) in 0.1 M NaOH (pH = 7.2) at a concentration of 1 wt%. Afterwards, the sample was incubated at room tempetature for 2 h and was then run electrophoretically for 1 h using 1 % agarose gel in TBE buffer at 100 V.

**DNA photocleavage assay.** The photo-induced DNA cleavage by **Ru_6_L** was examined by gel electrophoresis experiment. Supercoiled pEGFP DNA (0.5 μg) was treated with **Ru_6_L** at various +/- ratios in 50 mM Tris-HCl solution (pH = 7.4), and the samples were then irradiated at room temperature with Xe lamp (450 nm, 150 W). After irradiation, the samples were mixed with sodium dodecyl sulfate (SDS) at a concentration of 1 wt%. Afterwards, the samples were incubated at room temperature for 2 h and then were run electrophoretically for 1 h using 1 % agarose gel in TBE buffer at 100 V.

**Continuous irradiation in presence of DNA.** A continuous irradiation in presence of CT-DNA was performed with a mercury vapour lamp (Osram HBO 200 W) and a 2000 W quartz halogen lamp (Philips), cooled by water circulation. IR (water) and UV (KNO_2_) cut-off filters were inserted between the irradiation cell and the exciting source. All the experiments were performed with argon- and air- saturated solution (3 mL) containing **Ru_6_L** (5 μM) and CT-DNA (45 μM, bases).

| **Table S1** Photophysical data of **Ru_6_L** in DMSO/H_2_O (v/v = 1:99) at 298 K | | | | | | |
| --- | --- | --- | --- | --- | --- | --- |
| Complex | λ_ex_*^a^* | ε_ex_*^b^* | λ_em_*^c^* | φ*^d^* | τ/μs*^e^* | δ/GM*^f^* |
| **Ru_6_L** | 462 | 8.30 | 612 | 0.0270 | 0.107 | 175 |
| *^a^* λ_ex_ values of the one-photon absorption in nm. *^b^* Extinction coefficient in 1×10^4^ M^-1^×cm^-1^. *^c^* λ_em_ values of the one-photon emission spectra in nm. *^d^* Liminescence quantum yield. *^e^*Life time. *^f^* Two-photon absorption cross section at 830 nm measured in methanol. | | | | | | |


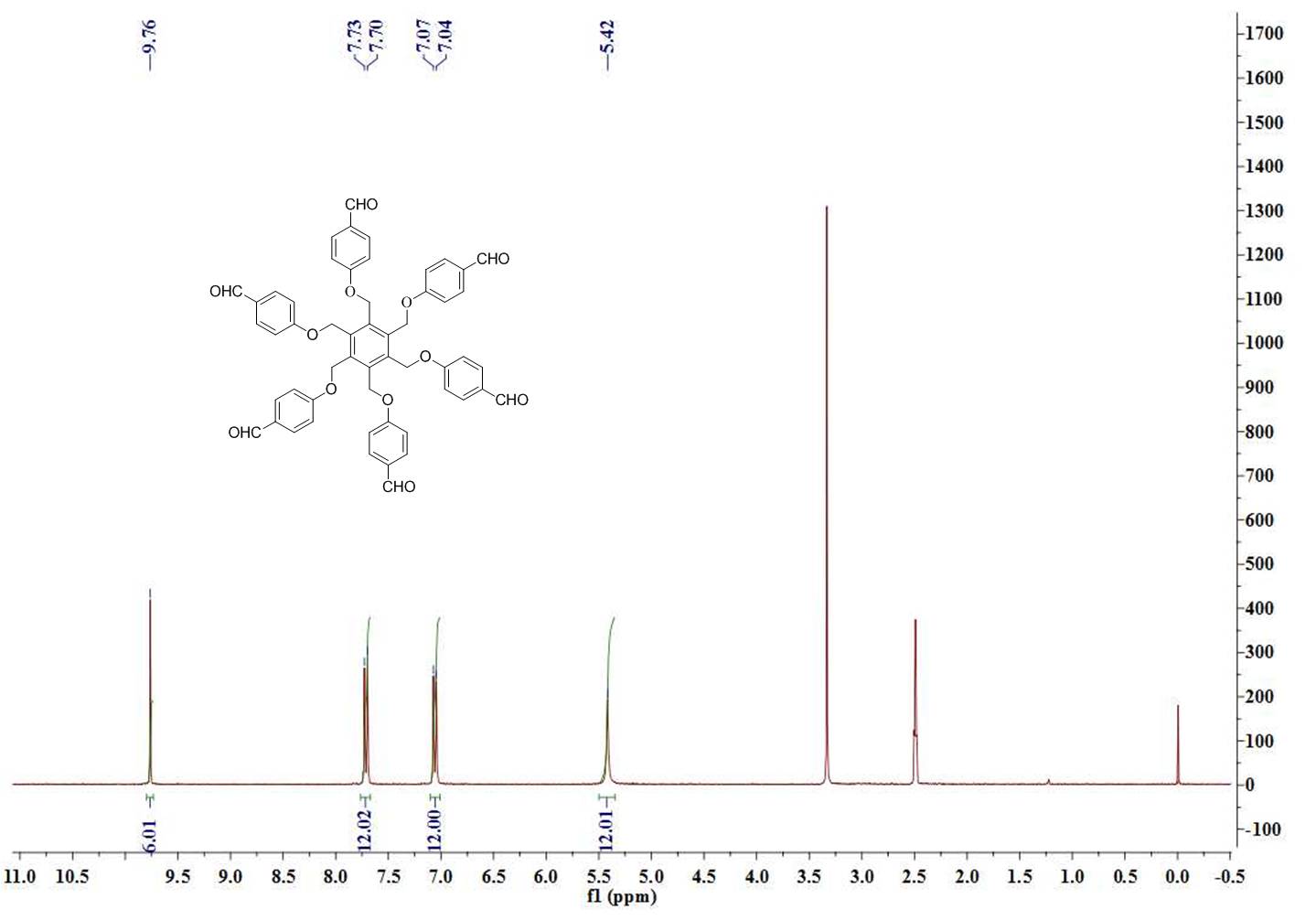


**Figure S1** ^1^H NMR spectrum of compound **Q**.


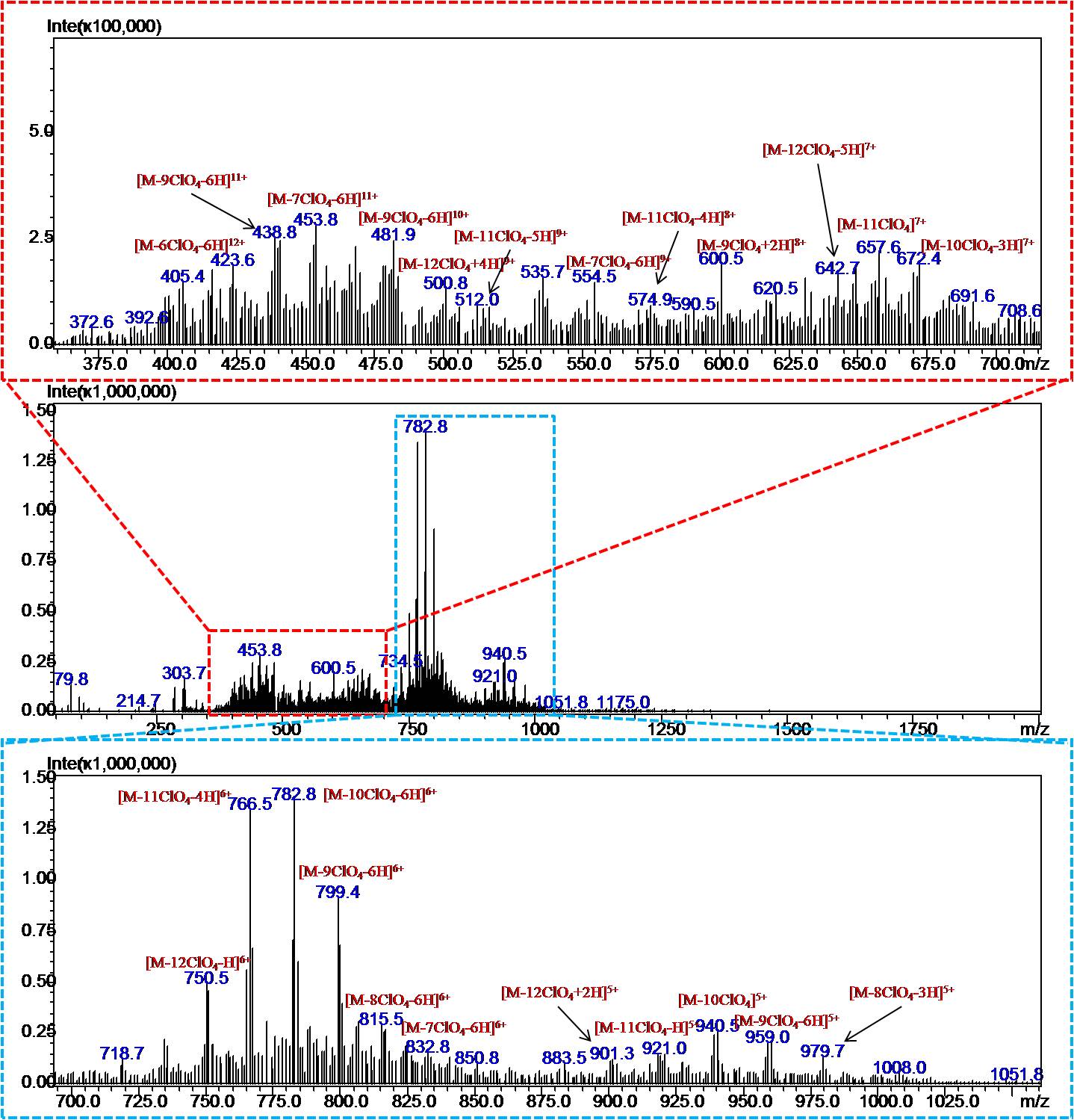


**Figure S2** ESI-MS spectrum of **Ru_6_L**.


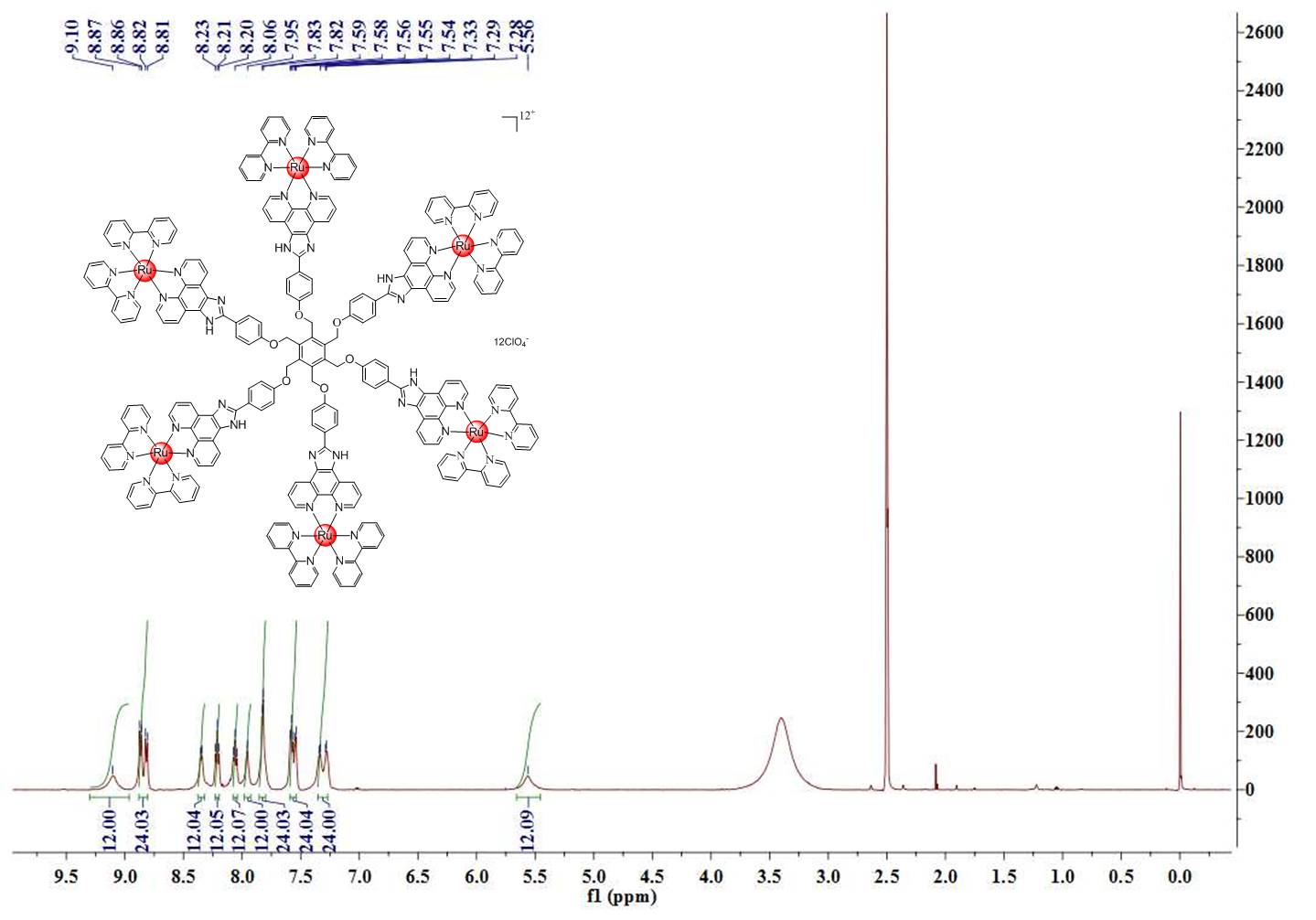


**Figure S3** ^1^H NMR spectrum of **Ru_6_L**.


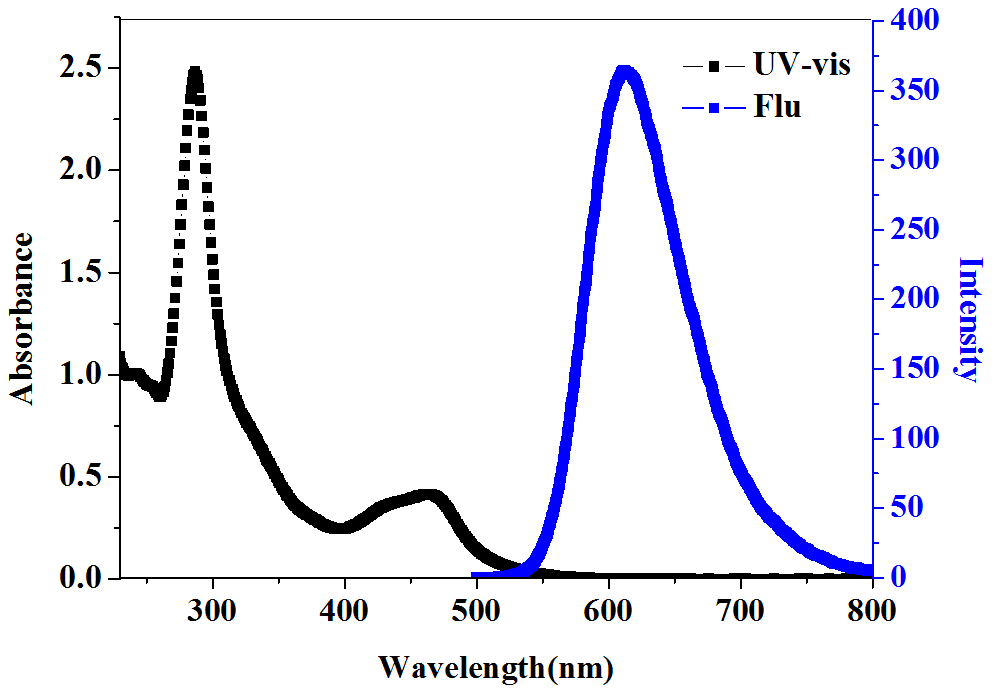


**Figure S4** Absorption spectra and emission spectra of **Ru_6_L** (5 μM).


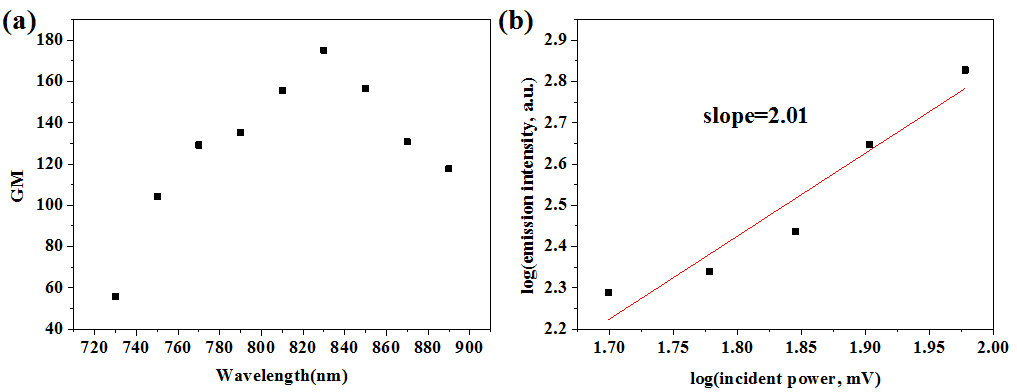


**Figure S5** (a) Two-photon absorption cross-sections of **Ru_6_L** at different exitation wavelength from 730 to 890 nm; (b) The logarithmic plots of the power dependence of relative two-photon induced luminescence intensity of **Ru_6_L** as a function of pump power at an excitation wavelength of 830 nm. The solid lines are the best-fit straight lines with gradient, slope=2.01, indicating that **Ru_6_L** is two-photon excitation active.


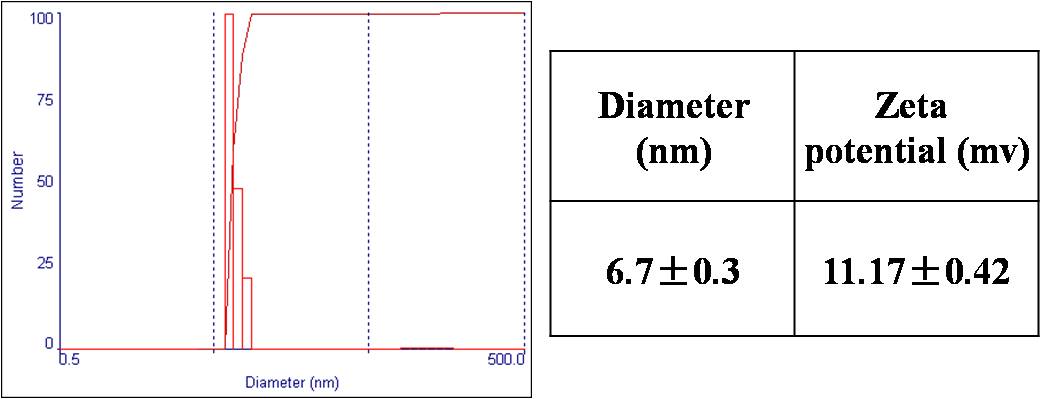


**Figure S6** Hydrodynamic diameter and zeta potential of complex **Ru_6_L** in aqueous solution determined by DLS.


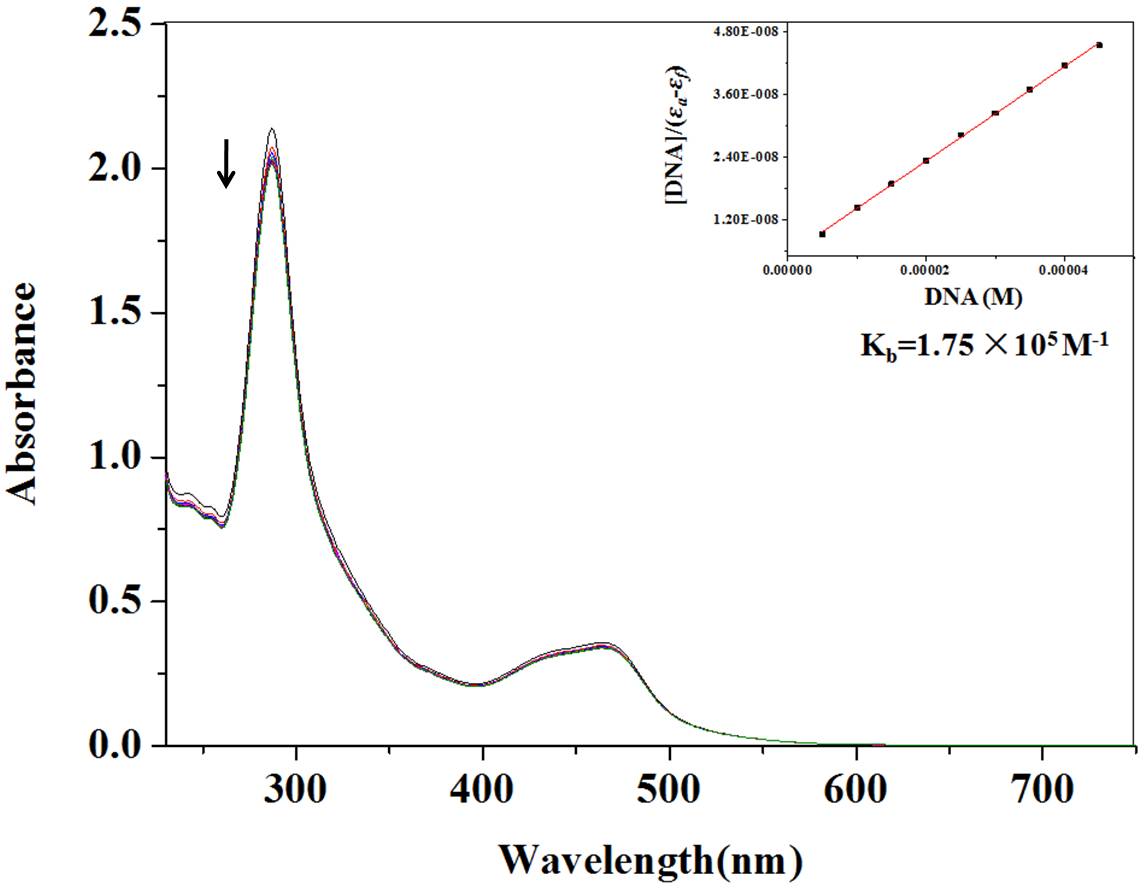


**Figure S7** Changes in absorption spectra of **Ru_6_L** (5μM) upon addition of CT-DNA (0-45 μM). Inset: plot of [DNA]/(𝜀_a_-𝜀_f_) vs [DNA] and the linear fit for the titration of the complex with the DNA.


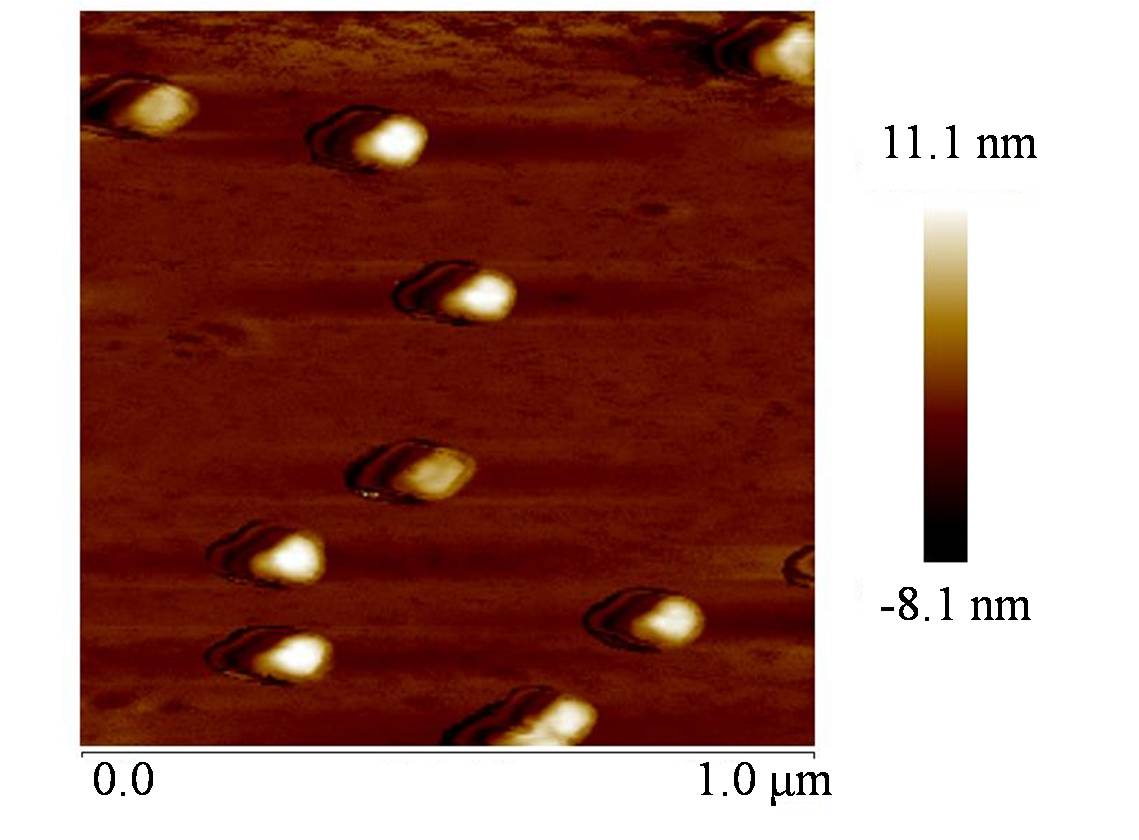


**Figure S8** AFM image of pBR 322 DNA (1.5 μM) condensation induced by incubation with **Ru_6_L** at the +/- ratio of 20.


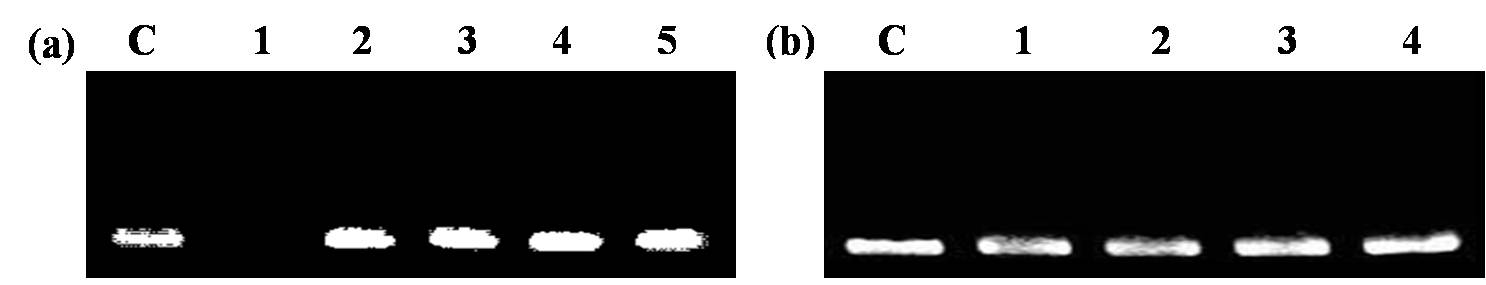


**Figure S9** (a) pBR 322 DNA protection from DNase-I enzyme by **Ru_6_L** at various +/- ratios in the prensence of DNase-I enzyme (Line 1-5). Naked DNA as the control (C); Lane 1: naked DNA; Line 2-5: the +/- ratios of 8, 12, 16 and 20. (b) Agarose gel of the photocleavage of pBR 322 DNA with **Ru_6_L** at the +/- ratios of 8, 12, 16 and 20 in air, respectively. Naked DNA as the control (C); Lane 1-4: the +/- ratios of 8, 12, 16 and 20.


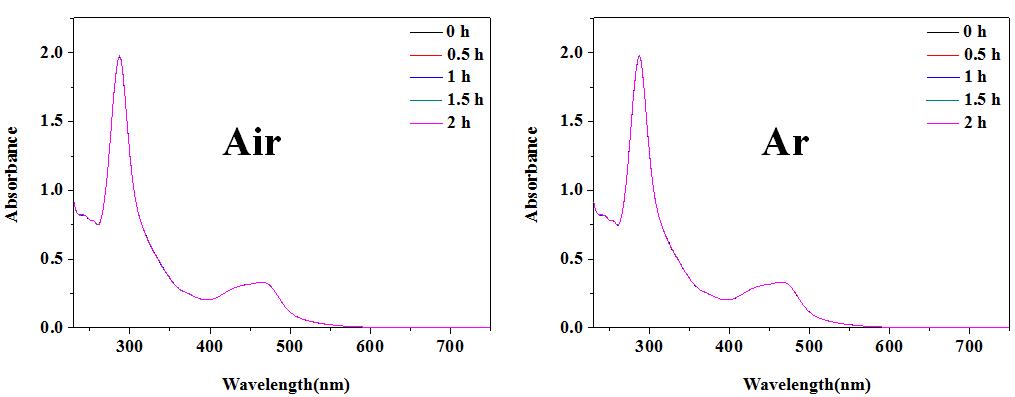


**Figure S10** Changes in absorption spectra of **Ru_6_L** (5 μM) under visible irradiation in the presence of CT-DNA (45 μM, base). Air means in air-saturated solution; Ar means in Argon-saturated solution. Irradiation time = 0, 0.5, 1, 1.5, 2 h.


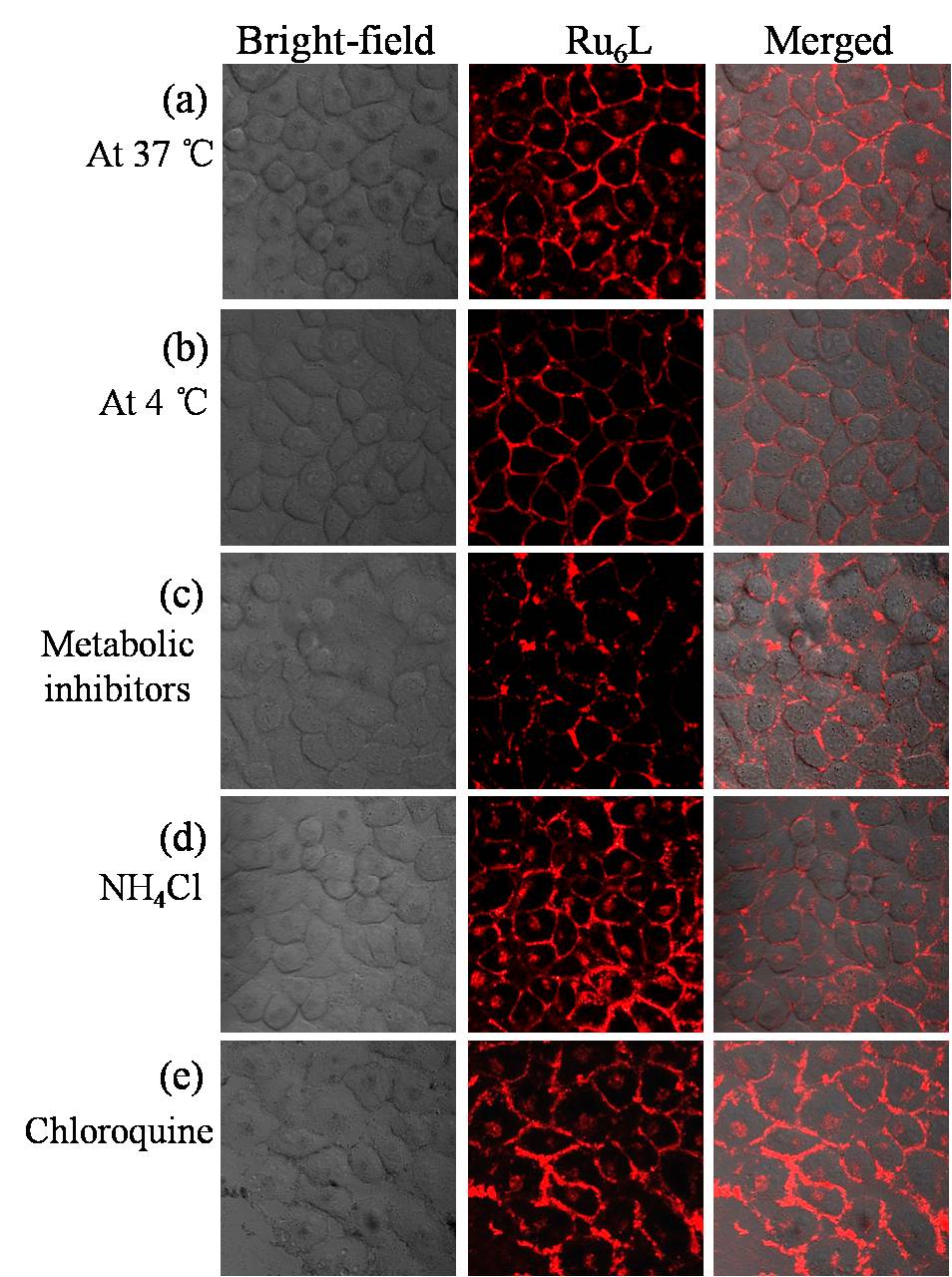


**Figure S11** TPM images of living HeLa cells incubated with 5 μM **Ru_6_L** under different conditions. (a) The cells were incubated with 5 μM **Ru_6_L** at 37 °C for 2 h. (b) The cells were incubated with 5 μM **Ru_6_L** at 4 °C for 2 h. (c) The cells were pretreated with 50 mM 2-deoxy-D-glucose and 5 μM oligomycin in PBS for 1 h at 37 °C and then incubated with 5 μM **Ru_6_L** at 37 °C for 2 h. (d and e) The cells were pretreated with endocytic inhibitors NH_4_Cl (50 mM), and chloroquine (50 μM) respectively, and then incubated with 5 μM **Ru_6_L** at 37 °C for 2 h.


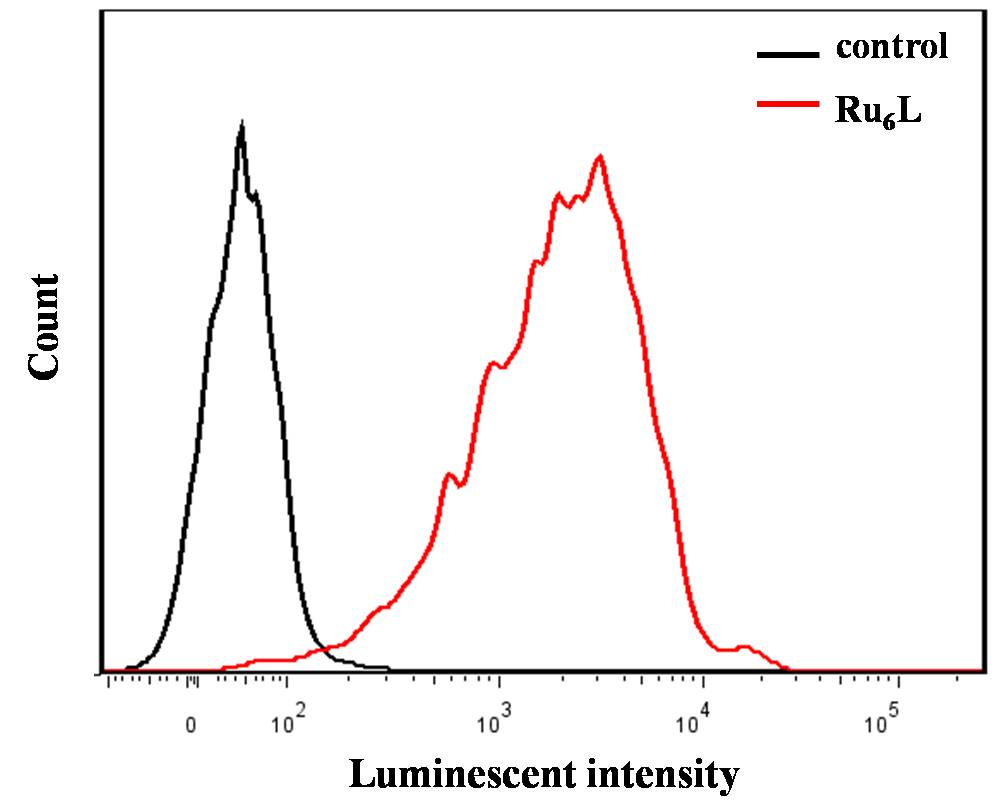


**Figure S12** Quantitative flow cytometry results on fluorescence intensities of HeLa cells incubation with **Ru_6_L**-pEGFP DNA particles for 4 h. The DNA concentration is 1.5 μM. **Ru_6_L**-DNA particles at the +/- ratio of 20.


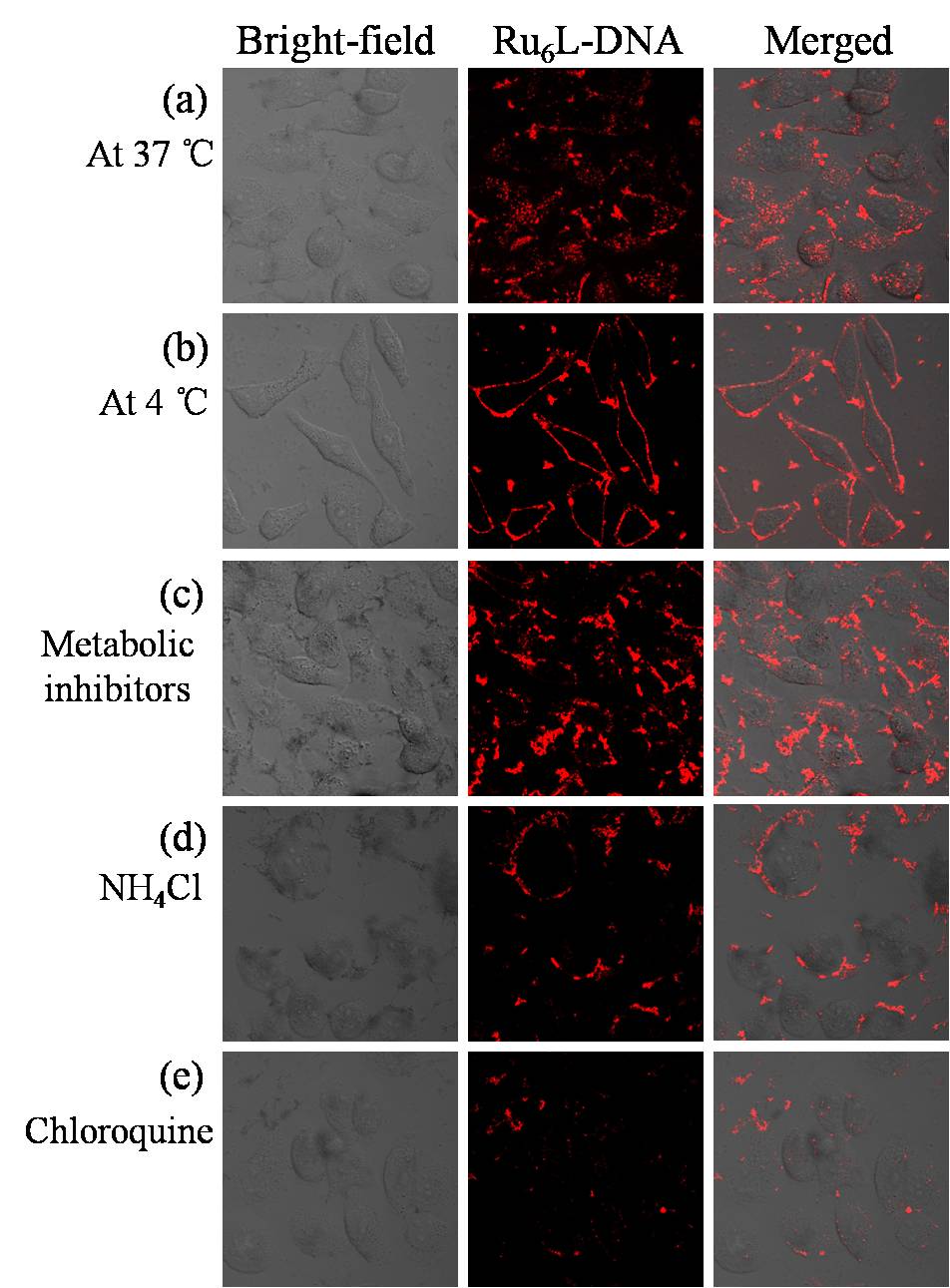


**Figure S13** TPM images of living HeLa cells incubated with **Ru_6_L**-pEGFP DNA particles under different conditions. (a) The cells were incubated with **Ru_6_L**-pEGFP DNA particles at the +/- ratio of 20 at 37 °C for 4 h. (b) The cells were incubated with **Ru_6_L**-pEGFP DNA particles at the +/- ratio of 20 at 4 °C for 4 h. (c) The cells were pretreated with 50 mM 2-deoxy-D-glucose and 5 μM oligomycin in PBS for 1 h at 37 °C and then incubated with **Ru_6_L**-pEGFP DNA particles at the +/- ratio of 20 at 37 °C for 4 h. (d and e) The cells were pretreated with endocytic inhibitors NH_4_Cl (50 mM), and chloroquine (50 μM) respectively, and then incubated with **Ru_6_L**-pEGFP DNA particles at the +/- ratio of 20 at 37 °C for 4 h.

**References:**

1. Marmur, J. A procedure for the isolation of deoxyribonucleic acid from micro-organisms. *J. Mol. Biol.* **3**, 208-218 (1961).
2. Reichmann, M. E., Rice, S. A., Thomas, C. A. & Doty, P. A further examination of the molecular weight and size of desoxypentose nucleic acid. *J. Am. Chem. Soc.* **76**, 3047-3053 (1954).
3. Wolf, A., Shimer, G. H. Jr. & Meehan, T. Polycyclic aromatic hydro- carbons physically intercalate into duplex regions. *Biochemistry* **26**, 6392-6397 (1987).
